# The Diversity of Serum Anti-DSG3 IgG Subclasses Has a Major Impact on Pemphigus Activity and Is Predictive of Relapses After Treatment With Rituximab

**DOI:** 10.3389/fimmu.2022.849790

**Published:** 2022-03-15

**Authors:** Marie-Laure Golinski, Alexandre Lemieux, Maud Maho-Vaillant, Marion Barray, Laurent Drouot, Damien Schapman, Marie Petit, Michael Hertl, Olivier Boyer, Sébastien Calbo, Pascal Joly, Vivien Hébert

**Affiliations:** ^1^ Normandie Univ, UNIROUEN, Inserm U1234, CHU Rouen, Department of Dermatology, Rouen, France; ^2^ Normandie Univ, UNIROUEN, Inserm U1234, Rouen, France; ^3^ Normandie Univ, UNIROUEN, PRIMACEN, Rouen, France; ^4^ Department of Dermatology and Allergology, Philipps University, Marburg, Germany; ^5^ Normandie Univ, UNIROUEN, Inserm U1234, CHU Rouen, Department of Immunology, Rouen, France

**Keywords:** desmoglein 3, immunoglobulin subclasses, pathogenicity, pemphigus, rituximab

## Abstract

**Introduction:**

We studied the distribution and *in vitro* pathogenicity of anti-DSG3 IgG subclasses during the course of pemphigus vulgaris (PV).

**Methods:**

We longitudinally studied the distribution of anti-DSG3 IgG subclasses (before *versus* after treatment) in sera from PV patients, using an addressable-laser bead immunoassay (ALBIA). The *in vitro* pathogenicity of corresponding sera was tested using keratinocyte dissociation and immunofluorescence assays.

**Results:**

Sixty-five sera were assessed at baseline (33 from patients treated with rituximab and 32 with corticosteroids). Sixty-three percent of these baseline sera contained 2 or more anti-DSG3 IgG subclasses *versus* 35.7% of sera from patients in complete remission (CR) and 75.0% of sera from patients with persistent disease activity after treatment. IgG4 was the most frequently detected anti-DSG3 IgG subclass, both in patients with disease activity and in those in CR. The presence of three or more anti-DSG3 IgG subclasses was predictive of relapse, in particular when it included IgG3, with a positive predictive value of 62.5% and a negative predictive value of 92%. While anti-DSG3 IgG4 Abs from sera collected before treatment were most often pathogenic, anti-DSG3 IgG4 from sera collected after treatment were pathogenic only after adjusting their titer to the one measured before treatment. The IgG3 fraction containing anti-DSG3 Abs also had an *in vitro* pathogenic effect. The disappearance of the pathogenic effect of some sera after removal of anti-DSG3 IgG3 suggested an additional effect of this IgG subclass.

**Conclusion:**

The serum levels and number of anti-DSG3 IgG subclasses drive the pathogenic effect of pemphigus sera and may predict the occurrence of relapses.

## Introduction

Pemphigus vulgaris (PV) is a potentially life-threatening autoimmune blistering disease caused by IgG autoantibodies (Abs) directed against desmoglein (DSG) 1 and DSG3 (1). The amount of IgG of anti-DSG Abs impairs desmosomal function. Moreover, recent data indicate that Abs engage signaling pathways interfering with different steps of desmosome turnover (2).

Clinically, the initial severity of pemphigus foliaceus (PF) and PV is correlated with anti-DSG1 and anti-DSG3 Ab serum levels, respectively (3–5). Moreover, the evolution of anti-DSG1 Abs, and to a lesser degree anti-DSG3 Abs, can predict the evolution of disease activity (6, 7). Indeed, while a re-increase or persistent high levels of anti-DSG1 Abs are closely correlated with the occurrence of skin relapses, anti-DSG3 Abs are less specific. In particular, anti-DSG3 Abs can occasionally be observed in some PV patients in clinical remission (5, 6, 8–11).

A preferential use of certain IgG subclasses has been identified in pemphigus patients. Classically, anti-DSG3 IgG4 Abs are predominantly found in PV sera from patients with active disease, followed by IgG1, and occasionally IgG2 and IgG3 (12–16). In humans, the IgG4 subclass is the smallest IgG fraction (<5%) and has paradoxically weak complement activation properties (17). The pathogenic effect of anti-DSG IgG4 Abs has been demonstrated in a PV mouse model (18), as well as in *in vitro* assays (19). Additionally, it has been demonstrated that anti-DSG IgG1 may also contribute to the pathogenic effect of pemphigus sera (20). Finally, the potential pathogenic effect of anti-DSG IgG2 and IgG3 subclasses has never been assessed in pemphigus.

The evolution of anti-DSG IgG subclasses according to patients’ clinical status has provided controversial results in the literature. While some studies reported a switch from IgG4 to IgG1 in patients in clinical remission (8, 21, 22), other studies did not find such results (16, 23).

We hypothesized that the level and distribution of anti-DSG3 IgG subclasses during the course of pemphigus may be implicated in the persistence of disease activity or achievement of clinical remission and might explain the paradoxical persistence of anti-DSG3 Abs in some patients in sustained clinical remission. Thus, we studied the distribution and the evolution of anti-DSG3 IgG subclasses in patients with PV using an addressable laser bead immuno assay (ALBIA) and correlated the distribution of anti-DSG3 IgG subclasses with the clinical course of patients included in the Ritux 3 trial. Finally, we analyzed the *in vitro* pathogenicity of corresponding sera using keratinocyte dissociation and immunofluorescence assays.

## Patients and Methods

### Population of Patients

We analyzed the sera from 33 and 32 PV patients assigned to the rituximab (RTX) and standard corticosteroid (CS) arms of the Ritux 3 trial, respectively, and for whom serum samples were available at baseline (24).

The longitudinal analysis was performed in 33 of these 65 PV patients (16 treated with RTX and 17 treated with CS) corresponding to those who had persistent anti-DSG3 Abs during the course of their disease as measured using the EUROIMMUN ELISA assay, whether or not they relapsed. Additionally, sera from 36 healthy donors (HD), 6 PF, 9 bullous pemphigoid (BP), and 5 PV patients with negative anti-DSG3 Abs after RTX treatment were used as negative controls.

### ALBIA of Anti-Dsg3 Abs and Their Subclasses

Sera from patients were analyzed before treatment at baseline, after treatment, and at the time of relapse, if applicable. To detect and quantify anti-DSG3 Ab IgG subclasses, we developed an ALBIA-DSG3, which consisted of coupling human recombinant DSG3 protein to fluorescent beads (LiquiChip Ni-NTA Beads; Qiagen, Hilden, Germany) according to the manufacturer’s protocol. To determine the isotype of serum anti-DSG3 Abs, DSG3-coated beads were incubated with sera diluted at 1:150, then incubated with anti-IgG1 (1:125), anti-IgG2 (1:125), anti-IgG3 (1:200), or anti-IgG4 (1:200) biotinylated secondary antibody (SouthernBiotech, Birmingham, AL, USA), and finally with streptavidin–R-phycoerythrin (Qiagen). The mean fluorescence intensity (MFI) was determined on a Bio-Plex apparatus using Manager software version 4.0 (Bio-Rad, Hercules, CA, USA). Negative control with no serum and positive control [anti-DSG3 Calibrator of ELISA kit (EUROIMMUN)] were included in every assay. The anti-DSG3 Ab serum levels were determined with the formula (MFI^serum^/MFI^Calibrator^) × 100, in which the calibrator was the anti-DSG3-positive control previously mentioned that was used on every 96-well plate and set arbitrarily to 100 arbitrary units (AU). For each isotype, we considered a positivity threshold corresponding to + 3 standard deviations relative to the mean value obtained from the sera of 36 HD.

### IgG Purification With ÄKTA-Start

Purification of IgG was performed by affinity chromatography, using ÄKTA-Start. The HiTrap Protein G column (GE Healthcare, Chicago, IL, USA) was equilibrated with 10 ml of phosphate-buffered saline (PBS) 1×, at pH 7.4. The 1/5 pre-diluted sera were added and IgG were eluted using glycine buffer 0.1 M, pH 2.7, followed by neutralization with 1 M Tris pH 9. Analyses were performed using UNICORN 7.0 software to collect IgG-containing fractions. Purified IgG were quantified using the BCA Protein Assay Kit (Pierce™, Rockford, IL, USA) according to the manufacturer’s instructions.

### Purification of IgG3

IgG3 purification was performed using the CaptureSelect™ IgG3 (Hu) Affinity Matrix (Thermo Scientific™, Rockford, IL, USA). The IgG3 column was equilibrated with 10 ml of phosphate-buffered saline (PBS) 1×, at pH 7.4. The 1/10 pre-diluted purified IgG were added, and flow without IgG3 was collected. IgG3 were then eluted using glycine buffer 0.1 M, pH 3, followed by neutralization with 1 M Tris pH 9. Purified IgG3 and flow without IgG3 were quantified using the BCA Protein Assay Kit (Pierce™) according to the manufacturer’s instructions.

### Keratinocyte Dissociation Assay

HaCaT cells were cultivated in 24-well plates with 600,000 cells per well in DMEM + GlutaMAX (Gibco, Grand Island, NY, USA) containing 1 mM CaCl_2_ in a humidified and controlled atmosphere (5% CO_2_) at 37°C. Twenty-four hours after reaching confluency, positive control AK23 (10 µg/ml), HD IgG (62.5 µg/ml), PV IgG (62.5 µg/ml), or IgG-depleted fractions collected from IgG-specific affinity purification (62.5 µg/ml) were added and incubated for 24 h. The amount of purified IgG used for the keratinocyte dissociation test of PV patients in CR whose sera contained only the anti-Dsg3 IgG4 subclass (before and after treatment) was adjusted to the level of anti-DSG3 Abs measured in the sera collected at baseline from the corresponding patients by a cross multiplication. Subsequently, the cells were treated with dispase solution (2.4 U/ml; Sigma-Aldrich, St. Louis, MO, USA) at 37°C until monolayers were released from plates. Monolayers were stained with crystal violet (Sigma-Aldrich) and subjected to mechanical stress by vigorously pipetting 7 times with a 1-ml pipette. Cell fragments were fixed, photos were taken from each well, and cell fragments were counted manually. All experiments were performed in triplicate.

### Immunofluorescence Assays

HaCaT cells were cultivated on 4-chamber Lab-Tek with 50,000 cells per cm^2^ in DMEM + GlutaMAX (Gibco) containing 10% fetal bovine serum and 1 mM CaCl_2_ per chamber until they reached at least 75% confluency. Cells were washed and incubated for 20 h with 62.5 µg/ml of HD IgG, PV IgG, or IgG-depleted fractions collected from IgG-specific affinity purification. Then, cells were fixed with 100% ethanol for 10 min, permeabilized with 0.3% Triton for 10 min, and washed after each step. Rat serum diluted at 1% was used for blocking. Cells were then incubated with a primary rabbit antibody anti-DSG3 coupled to Alexa-Fluor^®^ 647 (Santa Cruz, Dallas, TX, USA) for 90 min in the dark under slow agitation. Cells were finally washed and dried for 15 min, and one drop of mounting medium with DAPI (Invitrogen, Carlsbad, CA, USA) was added per condition. Photos were taken using a multiphoton confocal microscope Leica TCS SP8.

### Statistical Analyses

All statistical analyses were performed using GraphPad Prism (GraphPad Software, La Jolla, CA, USA). Determination of specificity and sensitivity of anti-DSG3 ALBIA and comparison of frequencies of anti-DSG3 subclasses were assessed using Fisher's exact test. Correlations were assessed using Pearson’s rank correlation coefficient. The numbers of anti-DSG3 IgG subclasses in relasping versus non-relapsing patients and the mean anti-DSG3 IgG4 serum level in patients with persistent active disease versus those in CR were compared using unpaired t-test. In PV patients with exclusively anti-DSG3 IgG4 Abs, the level of anti-DSG3 IgG4 at baseline and during the evolution was compared using a paired t-test. Differences were considered significant when p < 0.05.

## Results

### Patients and Times of Serological Assessment

**Figure 1:**
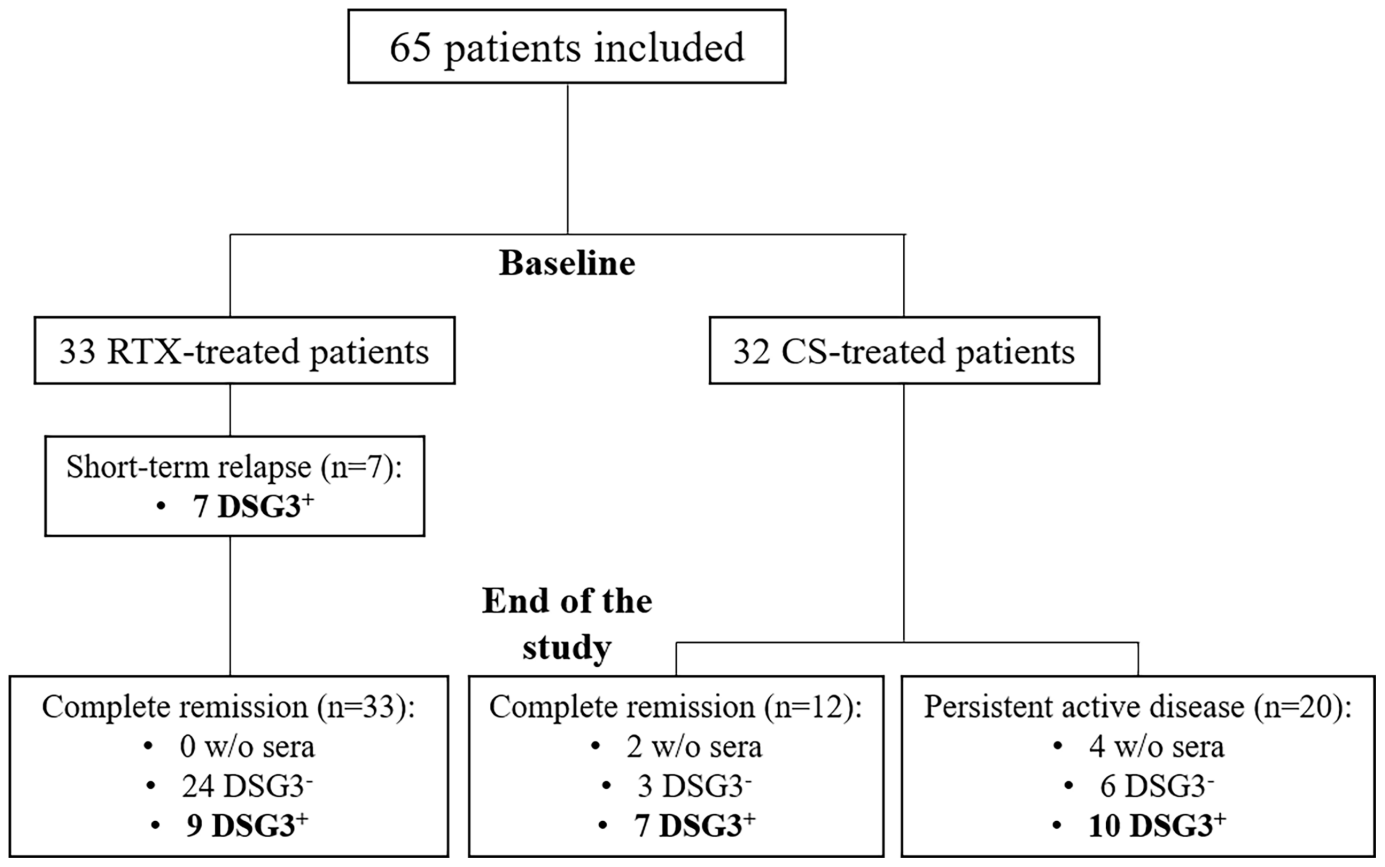
Flowchart of the study. CS, corticosteroids; DSG3, desmoglein 3; RTX, rituximab.

Sera from 65 patients were tested at baseline (33 treated with RTX and 32 treated with CS) (**Figure 1**).

A relapse occurred in 7 of 33 (21.2%) patients treated with RTX and in 20 of 32 (62.5%) treated with CS. The 7 patients who relapsed in the RTX group relapsed before Month 12 and were retreated with additional infusions of RTX. All of them were in complete remission (CR) at the Month 36 evaluation.

The distribution of anti-DSG3 IgG subclasses after treatment was performed in 2 subgroups of patients: i) those who relapsed or had persistent disease activity and ii) those who had a sustained CR. These latter patients were selected from the whole group of patients in CR after treatment on the fact that they had persistent positive serum anti-DSG3 Abs by ELISA, allowing to assess the distribution of anti-DSG3 Abs after treatment. Seventeen patients had a persistent disease activity after treatment, including the 7 patients who relapsed early after RTX, and 10 out of the 20 patients who relapsed after CS treatment.

The same serological analyses were performed at Month 36 after the start of treatment in 16 patients in sustained CR, including 7 treated with CS and 9 with RTX.

### Validation of the Anti-DSG3 ALBIA

We first assessed the specificity and sensitivity of our ALBIA for the detection of anti-DSG3 IgG subclasses. For this, we compared anti-DSG3 ALBIA performed with anti-DSG3 Abs containing sera from 65 PV patients with 56 control sera (36 HD, 6 PF, 5 PV with negative anti-DSG3 Abs after RTX treatment, and 9 BP). Our ALBIA had 92.31% sensitivity and 85.71% specificity for the detection of anti-DSG3 Abs (**Table 1**).

**Table 1:** Contingency table for determination of specificity and sensitivity of anti-Dsg3 ALBIA.

Anti-Dsg3 ALBIA			
	Positive	Negative	Total
PV patients	60	5	65
Negative controls	8	48	56
Total	68	53	121

**Figure 2:**
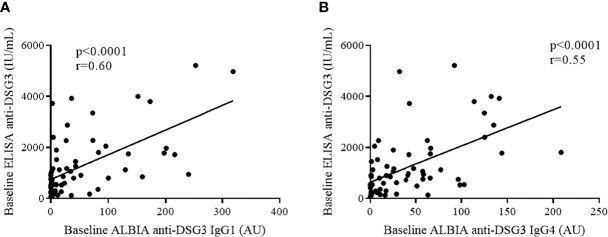
Diagnostic value of ALBIA in comparison with ELISA anti-DSG3 results in pemphigus patients at baseline (n = 65). Correlation between ELISA anti-DSG3 IgG and ALBIA anti-DSG3 IgG1 **(A)** or IgG4 **(B)** values was assessed using Pearson’s rank correlation coefficient. ALBIA, addressable laser bead immunoassay; AU, arbitrary units; DSG3, desmoglein 3; ELISA, enzyme-linked immunosorbent assay; IU, international unit.

Since IgG1 and IgG4 are the main anti-DSG3 IgG subclasses, we then correlated anti-DSG3 IgG1 and IgG4 serum levels measured by ALBIA with anti-DSG3 Ab serum levels measured by ELISA in sera from the same patients with active disease. A high correlation was observed for both IgG1 (r = 0.60, p < 0.0001) and IgG4 (r = 0.55, p < 0.0001) anti-DSG3 Abs (**Figure 2**).

### Distribution of Anti-DSG3 IgG Subclasses in PV Patients Before Treatment (Onset of Disease)

**Figure 3:**
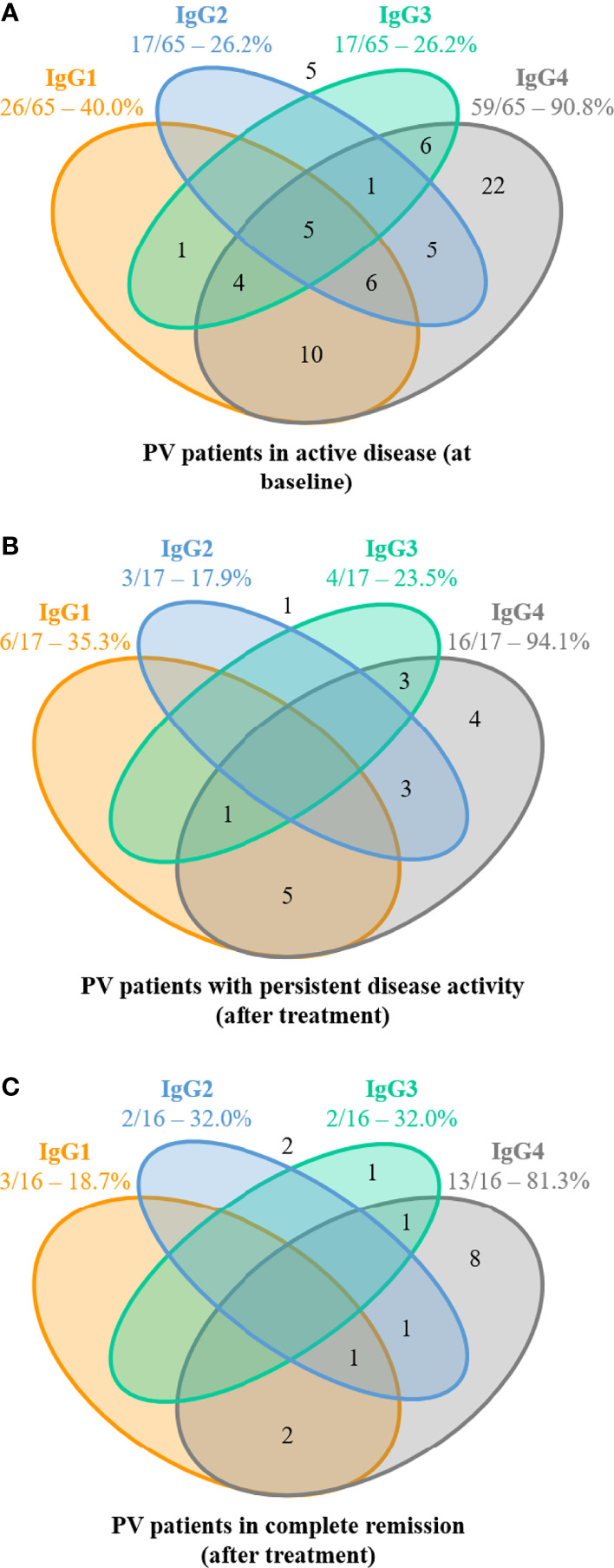
Distribution of anti-desmoglein 3 (DSG3) IgG subclasses from pemphigus patients with active disease (before treatment) and after treatment in patients with or without disease activity. **(A)** Distribution of anti-DSG3 IgG subclasses in the 65 pemphigus vulgaris (PV) patients with active disease at baseline. No anti-Dsg3 IgG subclasses were detected in 5 of these 65 patients (number shown outside). Among these 60 sera with positive ALBIA, 38 (63.3%) contained at least 2 or more anti-DSG3 IgG subclasses, while 22 (36.7%) sera contained only one anti-DSG3 IgG subclass (IgG4 in all cases). **(B)** Distribution of anti-DSG3 IgG subclasses in the 17 PV patients with persistent disease activity. No anti-Dsg3 IgG subclasses were detected in 1 of these 17 patients (number shown outside). Among these 16 sera with positive ALBIA, 12 sera (75.0%) still contained at least 2 or more anti-DSG3 IgG subclasses, while only 4 sera (25%) contained only one anti-DSG3 IgG subclass (IgG4 in all cases). **(C)** Distribution of anti-DSG3 IgG subclasses in the 16 PV patients in complete remission at Month 36. No anti-Dsg3 IgG subclasses were detected in 2 of these 16 patients (number shown outside). Among these 14 sera with positive ALBIA, only 5 sera (35.7%) still contained at least 2 or more anti-DSG3 IgG subclasses, while 9 sera (64.3%) contained only one anti-DSG3 IgG subclass (IgG4 in all but one case).

At baseline, anti-DSG3 IgG4 and IgG1 were detected in 90.8% and 40.0% of sera, respectively, whereas IgG2 and IgG3 were both detected in 26.2% of sera (**Figure 3A**).

Among the 60 patients with positive ALBIA, the most frequently detected isotypes of anti-DSG3 Abs were IgG4 either alone (36.7%) or most often combined with IgG1 (16.7%), IgG2 (8.3%), and IgG3 (10.0%). The combination of 3 or 4 IgG subclasses of anti-DSG3 Abs (including IgG4 in all cases) was observed in 26.6% of sera. Only one serum (1.7%) did not contain anti-DSG3 IgG4 Abs (**Figure 3A**).

### Evolution of the Distribution of Anti-DSG3 IgG Subclasses After Treatment

Among the patients with positive ALBIA, most sera (64.3%) from patients in CR contained only one anti-DSG3 IgG subclass (corresponding to IgG4 in all but one case), as compared with 25.0% of sera from patients with persistent disease activity, and 36.7% of baseline sera (**Figure 3**). In contrast, 35.7% of sera from patients in CR contained at least 2 or more anti-DSG3 IgG subclasses, as compared with 75.0% of sera from patients with persistent active disease and 63.3% of baseline sera (**Figures 3B, C**).

### Baseline Anti-DSG3 IgG Subclass Diversity as a Predictive Factor of Relapse

Since RTX is approved as a first-line treatment for pemphigus, we assessed whether the number and the isotypes of anti-DSG3 IgG subclasses in sera from patients at the onset of pemphigus might predict the occurrence of relapses under treatment or after treatment withdrawal.

**Figure 4:**
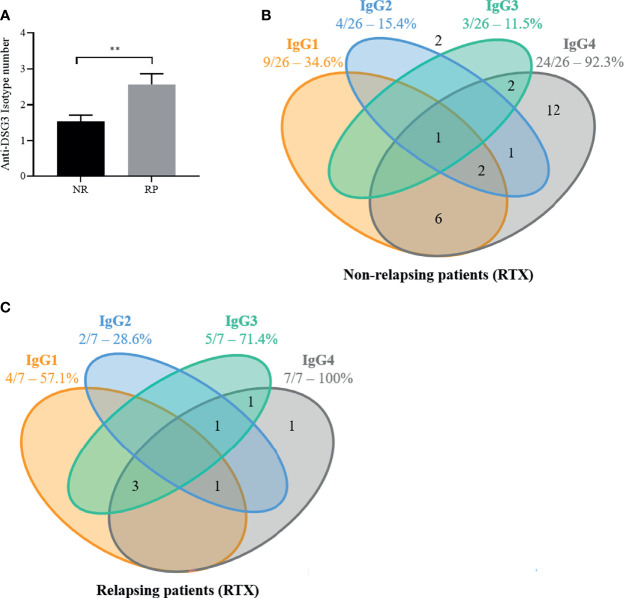
The number of anti-desmoglein 3 (DSG3) IgG subclasses as well as the presence of anti-DSG3 IgG3 in pemphigus vulgaris (PV) patients at baseline are associated with early relapse after rituximab (RTX) treatment. In the 33 PV patients treated by RTX, the distribution of anti-DSG3 IgG subclasses in the 26 non-relapsing (NR) **(A)** compared to the 7 early relapsing patients (RP) **(B)** show that RP have more IgG subclasses (p < 0.01) **(C)** and have a higher frequency of IgG3 than NR patients (71% vs. 12%; p = 0.004). No anti-Dsg3 IgG subclasses were detected in 2 of the 26 non-relapsing patients (number shown outside). Mean ± SEM were compared using unpaired t-test. Frequencies of anti-DSG3 subclasses were compared using Fisher’s exact test, **p < 0.01.

The mean number of anti-DSG3 IgG subclasses in the baseline sera was higher in patients who further relapsed than in patients who maintained a sustained clinical remission (2.6 ± 0.8 vs. 1.5 ± 0.9; p = 0.01) (**Figure 4A**). In particular, 5 of the 7 (71.4%) patients who relapsed had 3 or more anti-DSG3 IgG subclasses in their baseline serum *versus* 3 of the 26 (11.5%) patients who maintained a prolonged remission (p = 0.004), corresponding to a positive predictive value of 62.5% (95% CI 0.31–0.86) and a negative predictive value of 92% (95% CI 0.75–0.99) for the occurrence of relapses (**Figures 4B, C**).

Moreover, the occurrence of relapses was particularly frequent in patients whose baseline serum contained anti-DSG3 IgG3 Abs, since the baseline sera from 5 of the 7 relapsing patients (71.4%) contained anti-DSG3 IgG3 Abs *versus* 3 out of the 26 sera from non-relapsing patients (11.5%, p = 0.004) (**Figures 4B, C**).

The distribution of the other anti-DSG3 IgG subclasses (IgG1, IgG2, and IgG4) was not associated with the occurrence of relapse (p > 0.99, p = 0.39, and p = 0.58).

### Demonstration of the Pathogenic Activity of Anti-DSG3 IgG3 and IgG4 Subclasses

**Figure 5:**
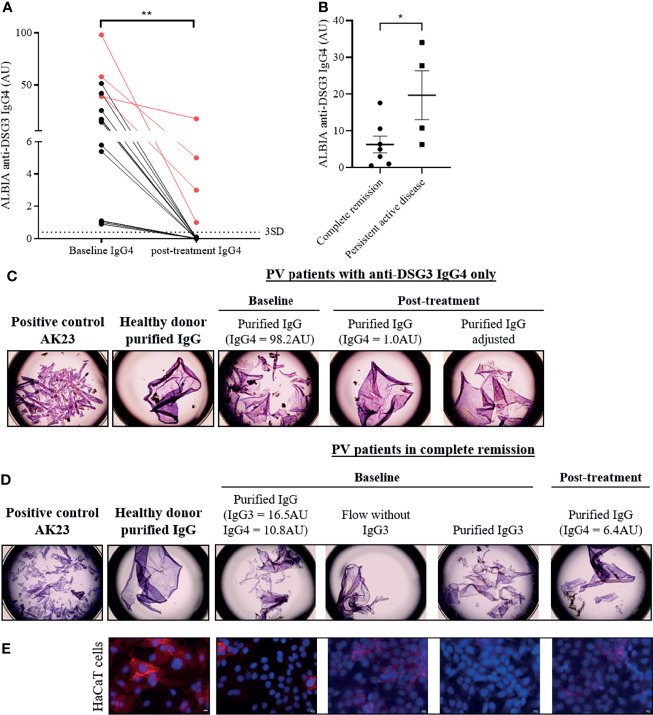
Pathogenicity of IgG3 and IgG4 anti-desmoglein 3 (DSG3) autoantibodies in pemphigus patients by keratinocyte dissociation and immunofluorescence assays. **(A)** IgG4 anti-DSG3 level (AU) were significantly decreased after remission (in RTX and CS groups) in pemphigus patients who had IgG4 only at baseline (n = 16) (p < 0.01). The four patients who still contained anti-DSG3 IgG4 after treatment are identified in red. The cutoff values are indicated as horizontal dotted lines and determined by the mean + 3 standard deviation (SD) of HD. Means were compared using paired t-test. **(B)** In patients whose sera contained exclusively anti-DSG3 IgG4 subclass after treatment, the mean anti-DSG3 IgG4 serum level in patients with persistent active disease was higher than in those in CR. Mean ± SEM were compared using unpaired t-test. **(C)** HaCaT cells pre-incubated for 24 h with positive control (AK23) or purified IgG of healthy donor (negative control) or PV patients with anti-DSG3 IgG4 only or **(D)** anti-DSG3 IgG3 and IgG4 were dissociated from the plate with dispase and the monolayers were mechanically disrupted. **(E)** HaCaT keratinocytes immunostaining incubated with purified IgG from a HD and from the patient’ IgG collected at the time of CR showed a linear labelling of DSG3 on the HaCaT cell plasma membrane. In contrast, the baseline purified IgG fraction induced the disappearance of the DSG3 labelling. Finally, removing the IgG3 fraction from the baseline patients’ serum led to the reappearance of the DSG3 staining, with a labelling close to that observed with control sera from HD, while the IgG3 fraction induced the disappearance of the DSG3 labelling. *p < 0.05; **p < 0.01.

Among the 16 patients who had exclusive anti-DSG3 IgG4 at baseline and did not further relapse, we selected 4 patients in sustained CR whose serum still contained anti-DSG3 IgG4. **Figure 5A** shows the decrease of anti-DSG3 IgG4 serum levels from these 4 patients (identified in red) relative to the 12 other sera which did not contain anti-DSG3 Abs anymore after treatment.

Interestingly, in patients whose sera contained anti-DSG3 IgG4 exclusively after treatment, the mean anti-DSG3 IgG4 Ab serum level in patients with persistent active disease was higher than in patients in CR (19.70 ± 13.26 AU *versus* 6.30 ± 6.06 AU; p = 0.044) (**Figure 5B**).

In agreement with this observation, **Figure 5C** shows that the serum collected at baseline (containing 98.2 AU of anti-DSG3 IgG4 by ALBIA) induced a strong keratinocyte dissociation, whereas the serum from the same patient collected at the time of CR (containing 1.0 AU of anti-DSG3 IgG4) did not induce a keratinocyte dissociation anymore. Interestingly, this latter serum recovered its pathogenic activity in keratinocyte dissociation assay after adjustment of the IgG4 level to the one at baseline (**Figure 5C**). In order to exclude the possible role of non-desmoglein Abs whose concentration could also have been increased after adjustment of the anti-DSG3 IgG titer, we previously checked that the sera collected from this patient both at baseline and at the time of CR did not contain anti-Dsc3 (desmocollin 3), anti-SPCA1 (secretory pathway Ca^2+^/Mn^2+^-ATPase isoform 1), or anti-CHRM3 (cholinergic receptor muscarinic 3) Abs, which have been reported to be involved in the pathogenicity of pemphigus patients (25, 26). These findings thus suggested that anti-DSG3 IgG4 collected at the time of CR were pathogenic, but the amount of antibody was not sufficient to induce *in vitro* keratinocyte dissociation.

We then tested a serum which contained at baseline a combination of anti-DSG3 IgG3 and IgG4 at rather low levels (IgG3: 16.5 AU; IgG4: 10.8 AU) and contained only IgG4 at the time of CR after treatment (6.4 AU). Purified IgG from the baseline serum but not from the serum collected at the time of CR induced a keratinocyte dissociation, while both sera contained rather close levels of anti-DSG3 IgG4 Abs (10.8 AU and 6.4 AU, respectively). Interestingly, when removing IgG3 Abs from the baseline serum, the purified IgG did not induce a keratinocyte dissociation anymore, whereas the purified IgG3 fraction alone did (**Figure 5D**), suggesting the role of the anti-DSG3 IgG3 subclass in the pathogenic activity of this patient’ serum, in addition to IgG4.

We then tested this latter serum using an immunofluorescence assay to further assess its pathogenic activity. **Figure 5E** shows that, in contrast with the purified IgG from a HD and from the patient’ IgG collected at the time of CR which both showed a linear labeling of DSG3 on the HaCaT cell plasma membrane, the baseline purified IgG fraction which contained a combination of anti-DSG3 IgG3 and IgG4 induced the disappearance of the DSG3 labeling, confirming the pathogenic activity of this baseline serum. Finally, removing the IgG3 fraction from the baseline patients’ serum led to the reappearance of the DSG3 staining, with a labeling close to that observed with control sera from HD, while the IgG3 fraction induced the disappearance of the DSG3 labeling (**Figure 5E**).

## Discussion

This study shows that while anti-DSG3 IgG4 were detected in more than 90% of pemphigus sera at baseline, this IgG4 subclass was also present in sera from all but one patient in sustained CR who still had positive circulating anti-DSG3 Abs. The main evolution in anti-DSG3 IgG subclasses that we observed during the follow-up of patients is that while 61% of sera collected at the onset of disease and 75% of sera from relapsing patients contained two or more IgG subclasses (including IgG1, IgG2, and IgG3 in 40%, 26%, and 26% of sera, respectively), in contrast, 64.3% of sera collected in patients in CR had only one anti-DSG3 IgG subclass, mainly corresponding to IgG4.

It has been suggested that the absence of pathogenic activity of pemphigus sera, which still contained anti-DSG3 Abs, was related to a switch of anti-DSG3 IgG subclasses from IgG4 to IgG1 (8, 21). We did not observe such an evolution, since anti-DSG3 IgG4 Abs were the exclusive IgG subclass (with no other anti-DSG3 IgG subclass) detected in the majority of sera (57.1%) from patients in CR. In contrast, we observed a close correlation between the clinical status (active disease *versus* remission) and the level of anti-DSG3 IgG4 Abs, whose mean level was 5.56 AU in patients in CR *versus* 19.70 AU in patients with persistent active disease (p = 0.026), suggesting that the level of anti-DSG3 IgG4 Abs rather than a switch in anti-DSG3 IgG subclasses is involved in disease activity (27–29). In accordance with these findings, we showed that the *in vitro* pathogenic activity of sera collected in patients in CR, which exclusively contained anti-DSG3 IgG4, was highly dependent upon the level of anti-DSG3 IgG4. In particular, we showed that the adjustment of anti-DSG3 IgG4 level to the one at baseline restored the pathogenic activity of a serum collected in a patient in CR, as recently reported (30). This observation might explain the persistence of anti-DSG3 Abs in some patients in CR, and the poor specificity (between serum level and disease activity) of DSG3 ELISA assays (6, 11). Nevertheless, although we confirmed that the patient serum did not contain anti-Dsc3, anti-SPCA1, or anti-CHRM3 Abs (which have been reported to be involved in the pathogenicity of pemphigus serum (25, 26)), we cannot completely exclude that the adjustment of the anti-DSG3 IgG4 titers could have induced an increased concentration of other pathogenic non-DSG Abs.

It is likely that the combination of multiple anti-DSG3 IgG subclasses may be involved in the pathogenic activity of patients’ sera. This is first suggested by the fact that most sera (63.3%) collected at the onset of pemphigus or in patients with persistent disease activity under treatment (75%) contained two or more anti-DSG3 IgG subclasses, while on the contrary, most sera (64.3%) from patients in CR contained only one anti-DSG3 IgG subclass (corresponding to IgG4 in all but one case). In addition, we showed that a baseline serum, which contained both anti-DSG3 IgG3 and IgG4 Abs, induced a keratinocyte dissociation, whereas a serum collected in the same patient in CR, which contained exclusively anti-DSG3 IgG4 at a level close to that at baseline, had no pathogenic activity. The persistence of a high number of anti-Dsg3 IgG subclasses in patients with persistent disease activity could be related to inadequate depletion of plasma cell populations expressing various isotypes and thus Abs directed against multiple epitopes. We previously showed that RTX induced a significant decrease of IgG-switched DSG-specific and non-specific memory B cells, and a disappearance of anti-DSG antibody-secreting cells, which were no longer detected in patients in complete remission after RTX. In contrast, CS did not modify the frequency or the phenotype of DSG-specific and non-specific memory B cells, and anti-DSG antibody-secreting cells were still detected after treatment, even in patients in remission (31). These findings are in agreement with the evolution of the distribution of anti-DSG3 IgG subclasses that we observed after RTX and CS treatment.

In accordance with the high clinical activity of RTX compared to a standard CS regimen, we observed a 1.6-fold decrease in the mean number of anti-DSG3 IgG subclasses from 1.8 at the onset of disease to 1.1 in patients treated with RTX, whereas the number of anti-DSG3 IgG subclasses did not change much in patients treated with the standard CS regimen (1.9 *versus* 1.7).

As a result of these previous findings, we assessed whether the number of anti-DSG3 IgG subclasses and their isotypes in baseline sera might predict the occurrence of persistent disease activity or relapse under treatment. We observed that the mean number of anti-DSG3 IgG subclasses in the baseline sera was higher in patients who further relapsed than in patients who maintained a sustained remission (2.6 ± 0.8 vs. 1.5 ± 0.9; p = 0.01), corresponding to a positive predictive value of 62.5% and a negative predictive value of 92%. Interestingly, relapses were particularly frequent in patients whose baseline serum contained anti-DSG3 IgG3 Abs, since anti-DSG3 IgG3 Abs were detected at baseline in sera from 71.4% of patients who further relapsed, as compared with 11.5% of patients who maintained CR (p = 0.004). In accordance with these findings, we showed that the IgG3 fraction had an *in vitro* pathogenic effect, and that its removal from the baseline serum, which contained a rather low level of anti-DSG3 IgG4, removed the pathogenic activity of this serum.

Overall, our findings should help physicians in the management of pemphigus patients. Indeed, taking into account the isotype(s) of anti-DSG3 IgG subclasses might help physicians to better predict the patients with a high risk of relapse. Our findings also raise the question of the epitopes recognized by the different IgG subclasses on the DSG3 protein. In particular, it might be hypothesized that the different anti-DSG3 IgG subclasses might target different epitopes on DSG3, thus promoting the pathogenicity of anti-DSG3 Abs, as suggested by Cho et al. (10).

## Data Availability Statement

The raw data supporting the conclusions of this article will be made available by the authors, without undue reservation.

## Ethics Statement

The studies involving human participants were reviewed and approved by the ethics committee from Normandie. The patients/participants provided their written informed consent to participate in this study.

## Author Contributions

M-LG, PJ, and VH wrote and conceived the manuscript. M-LG, AL, MM-V, MB, LD, DS, MP, MH, and VH generated and analyzed the data. OB, SC, PJ, and VH revised the manuscript. All authors contributed to the article and approved the submitted version.

## Funding

This study was supported by INSERM, Normandy University, and Rouen University Hospital, Dermatology Department, France.

## Conflict of Interest

The authors declare that the research was conducted in the absence of any commercial or financial relationships that could be construed as a potential conflict of interest.

## Publisher’s Note

All claims expressed in this article are solely those of the authors and do not necessarily represent those of their affiliated organizations, or those of the publisher, the editors and the reviewers. Any product that may be evaluated in this article, or claim that may be made by its manufacturer, is not guaranteed or endorsed by the publisher.
